# Supplementary figures and images for: Structural modeling of the flagellum MS ring protein FliF reveals similarities to the type III secretion system and sporulation complex
Source: PeerJ. 2016 Feb 22;4:e1718. doi: 10.7717/peerj.1718 (PMC4768692; doi:10.7717/peerj.1718)

*Bacillus subtilis*

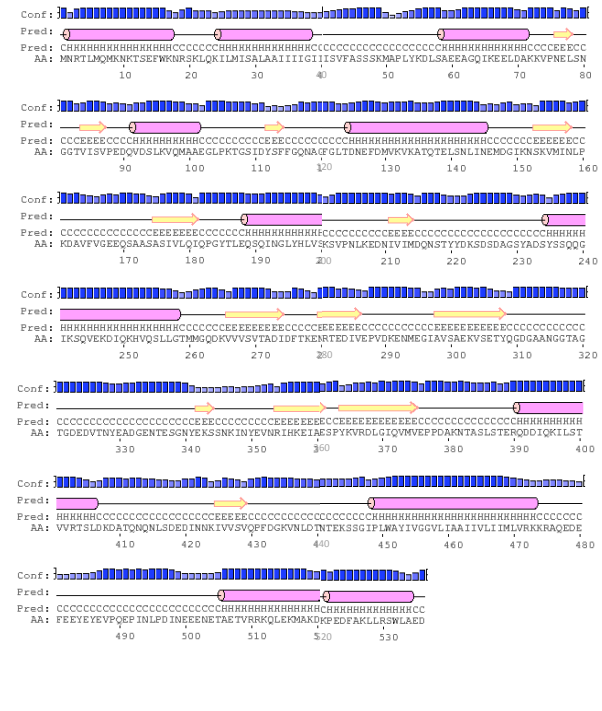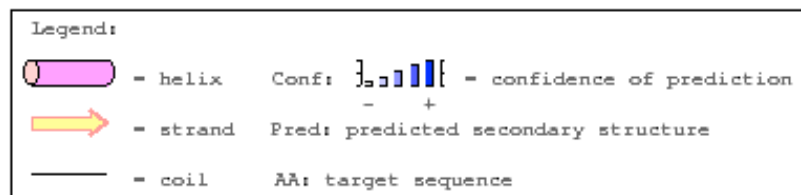

Supplement: Figure S1 — The secondary structure prediction generated by the PSIPRED server is shown for the S. typhiomurium, T. pallidum and B. subtilis FliF sequences. [file peerj-04-1718-s001.pdf]
