# Supplementary material for: Structural modeling of the flagellum MS ring protein FliF reveals similarities to the type III secretion system and sporulation complex
Source: PeerJ. 2016 Feb 22;4:e1718. doi: 10.7717/peerj.1718 (PMC4768692; doi:10.7717/peerj.1718)
Supplement: Figure S2 — The top scoring template(s) identified by the threading server Phyre are shown for three different regions of FliF RBM3. [file peerj-04-1718-s002.pdf]

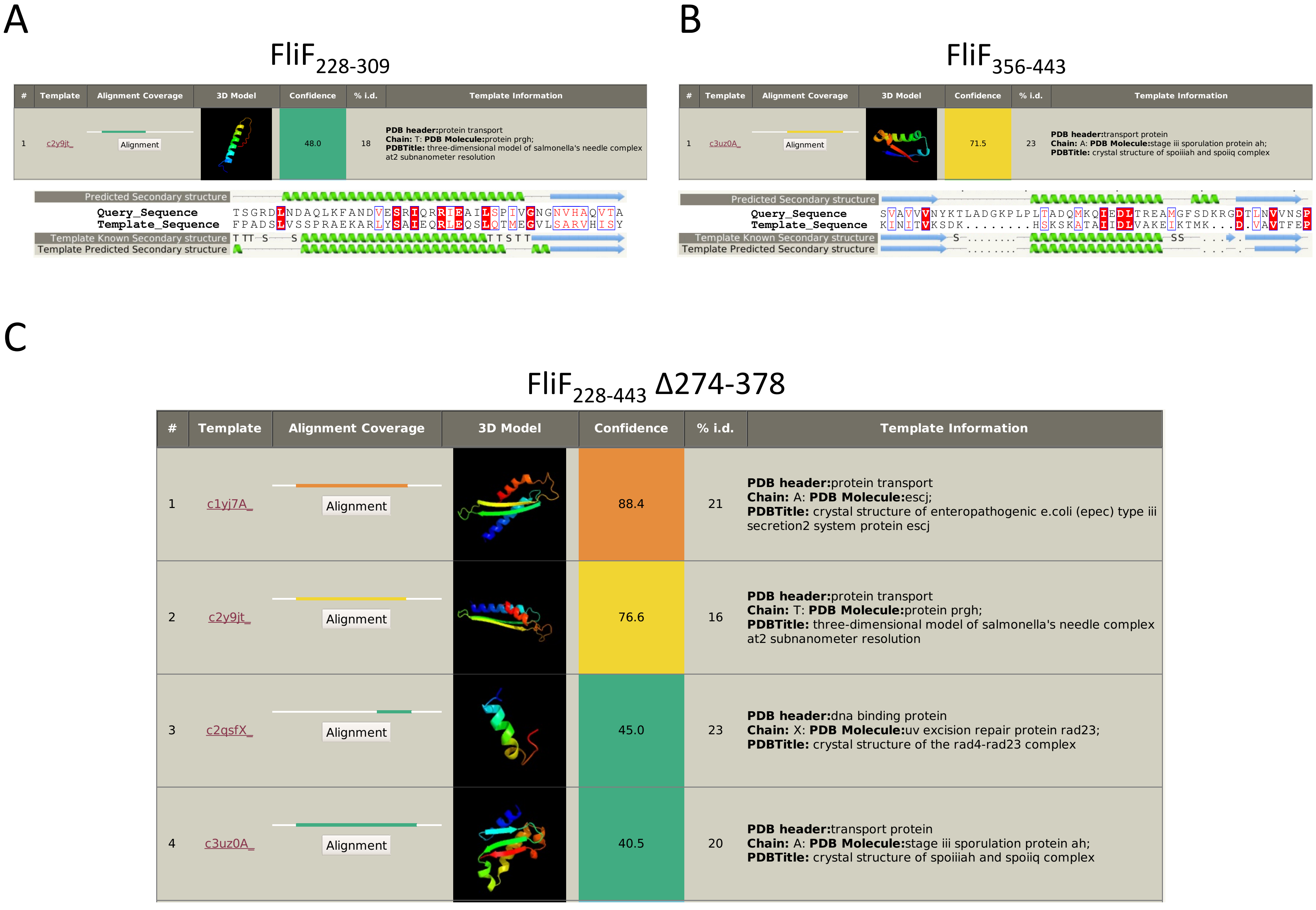
A
FliF228-309
| Template | Alignment Coverage | 3D Model | Confidence | % i.d. | Template Information
1 | c2y9jt_ | Alignment | | 48.0 | 18 | PDB header:protein transport Chain: T: PDB Molecule:protein prgh; PDBTitle: three-dimensional model of salmonella's needle complex at2 subnanometer resolution
Predicted Secondary structure
Query_Sequence TSGRDLNDAQLKFANDVESRIQRRIEAILSPIVGNGNVHAQVTA
Template_Sequence FPADSLVSSPRAEKARLYSAIEQRLEQSLQTMEGVLSARVHISY
Template Known Secondary structure T TT S S TTSTT
Template Predicted Secondary structure
B
FliF356-443
| Template | Alignment Coverage | 3D Model | Confidence | % i.d. | Template Information
1 | c3uz0A_ | Alignment | | 71.5 | 23 | PDB header:transport protein Chain: A: PDB Molecule:stage iii sporulation protein ah; PDBTitle: crystal structure of spoiiiah and spoiiq complex
Predicted Secondary structure
Query_Sequence SVAVVVNYKTLADGKPLPLTADQMKQIEDLTREAMGFSDKRGDTLNVVNSP
Template_Sequence KINITVKSDK........HSKSKATAIIDLVAKEIKTMK...D.VAVTFEP
Template Known Secondary structure S........ SS ... .
Template Predicted Secondary structure ....
C
FliF228-443 Δ274-378
| Template | Alignment Coverage | 3D Model | Confidence | % i.d. | Template Information
1 | c1yj7A_ | Alignment | | 88.4 | 21 | PDB header:protein transport Chain: A: PDB Molecule:escj; PDBTitle: crystal structure of enteropathogenic e.coli (epec) type iii secretion2 system protein escj
2 | c2y9jt_ | Alignment | | 76.6 | 16 | PDB header:protein transport Chain: T: PDB Molecule:protein prgh; PDBTitle: three-dimensional model of salmonella's needle complex at2 subnanometer resolution
3 | c2qsfX_ | Alignment | | 45.0 | 23 | PDB header:dna binding protein Chain: X: PDB Molecule:uv excision repair protein rad23; PDBTitle: crystal structure of the rad4-rad23 complex
4 | c3uz0A_ | Alignment | | 40.5 | 20 | PDB header:transport protein Chain: A: PDB Molecule:stage iii sporulation protein ah; PDBTitle: crystal structure of spoiiiah and spoiiq complex
