# Supplementary material for: Structural modeling of the flagellum MS ring protein FliF reveals similarities to the type III secretion system and sporulation complex
Source: PeerJ. 2016 Feb 22;4:e1718. doi: 10.7717/peerj.1718 (PMC4768692; doi:10.7717/peerj.1718)
Supplement: Supplementary Methods — Flags and commands used for the EM-guided symmetry modeling procedure—See Materials and Methods [file peerj-04-1718-s003.docx]

EM-guided symmetry modeling procedure:

--------------------------------------------------------

Execute Rosetta:

~rosetta/rosetta_score/bin/minirosetta.macosx

Using the following flags:

-run:protocol symdock

-database ~rosetta/rosetta_database

-in:file:s input.pdb

-symmetry:symmetry_definition 25.symm

-symmetry:initialize_rigid_body_dofs

-edensity:mapfile FliF_map.mrc

-edensity:mapreso 22.0

-edensity:grid_spacing 5.0

-edensity:score_symm_complex true

-packing:ex1

-packing:ex2aro

-use_input_sc

-ignore_unrecognized_res

-out:nstruct 1000

-out:file:silent phaseI.silent

-out:file:silent_struct_type binary

-out:file:fullatom

-residues:patch_selectors CENTROID_HA

-use_incorrect_hbond_deriv false

-docking:low_patch patch_phaseI

-docking:high_patch patch_phaseI

-docking:high_min_patch patch_high_min_phaseI

-docking:pack_patch patch_phaseI

-docking:dock_lowres_filter 15.0 20.0 1500.0

Content of file patch_phaseI:

elec_dens_whole_structure_ca = 0.1

Content of file patch_high_min_phaseI:

elec_dens_whole_structure_ca = 0.1

fa_rep *= 4.22

24-fold, 25-fold and 26-fold symmetry files were generated by manually placing the corresponding number of subunits in the FliF EM map, using the following command:

perl $rosettadir/rosetta_source/src/apps/public/symmetry/make_symmdef_file.pl -a A -i B -r 12.0 \

–p input.pdb > input.symm
